# Factors associated with different smoking statuses among Malaysian adolescent smokers: a cross-sectional study

**DOI:** 10.1186/s12889-019-6857-3

**Published:** 2019-06-13

**Authors:** A. H. Nur Atikah, Lei Hum Wee, M. S. Nur Zakiah, Caryn Mei Hsien Chan, N. M. Mohamed Haniki, J. S. Swinderjit, Ching Sin Siau

**Affiliations:** 1 Health Education Program, Community Health Centre, Faculty of Health Sciences, The National University of Malaysia (UKM), Kuala Lumpur, Malaysia; 2 Kulliyah of Pharmacy, International Islamic of Malaysia (UIA), Kuala Lumpur, Malaysia; 3 National Cancer Society Malaysia (NCSM), Kuala Lumpur, Malaysia; 4 Faculty of Social Sciences and Liberal Arts, UCSI University, Kuala Lumpur, Malaysia

**Keywords:** Socioeconomic, Smoking status, Preference, Adolescents, Malaysia

## Abstract

**Background:**

This study focused on the associations between socioeconomic status (SES) and adolescent smoking among secondary school students (13 to 17 years) in the Federal Territory of Kuala Lumpur, Malaysia. Our objective was to evaluate the relationships between adolescent demographics, socioeconomic status and smoking status.

**Methods:**

The survey data were based on baseline findings from a cross-sectional study (*N* = 422 adolescents). Chi-square test was used to assess the relationship between demographic characteristics, socioeconomic status (household monthly income and daily allowance) and adolescent smoking status. Exhaled carbon monoxide (CO) reading and the Hooked on Nicotine Checklist (HONC) were used to evaluate adolescent smoking status. A Multivariate Multinomial Logistic Regression (MMLR) was employed to test selected demographic and socioeconomic predictors of smoking status.

**Results:**

Of the 422 adolescents (*M* age = 15.58, *SD* = 1.24), more than half of the participants initiated smoking between 13 to 17 years old (59.0%). A total of 308 (73.0%) were electronic cigarette users, with more than 50% comprising of single users. The mean CO reading was 2.14 ppm with 78.0% of adolescents scoring more than 0 on the Hooked on Nicotine Checklist (HONC). Males and participants aged 15 and 16 years were at increased risks of sole CC smoking. Meanwhile, males, those who are not hooked on smoking and with a non-smoker CO reading were at increased risks of sole EC smoking. Finally, Bumiputeras were at less risk of EC smoking.

**Conclusions:**

Demographic variables such as age, gender and ethnicity predicted smoking status predicted smoking risk, but not socioeconomic factors. The findings allow policy makers to target specific high-risk demographic groups when designing smoking cessation programs for adolescents.

## Background

Adolescence marks a critical growth period, with many lifetime health behaviors formed during this time. This is a crucial stage for the development of health behaviors such as smoking and the habits that develop during this stage of life are often carried over into adulthood [1–3]. Rapid physical, social, emotional and cognitive development during this period of development is typically characterized by curiosity and smoking experimentation/initiation [3]. Thus, targeting the prevention of smoking at this juncture may be key to the reduction of many preventable chronic diseases in adulthood. The need and urgency of this issue takes on new meaning with the high prevalence of adolescent smoking in Malaysia, where 1 in 10 adolescents in the 13 to 17-year-old age group are smokers [4]. Examining the sociodemographic characteristics of adolescent smoking is the key step toward its prevention.

In order to intervene with adolescent smoking, there is a need to grasp the rapidly changing landscape of adolescent tobacco use, which is marked by recent decreases in combustible cigarette smoking and overall increases in the use of electronic cigarettes (EC) and other nicotine delivery systems such as shisha [5–9]. A study on EC use among Malaysian adolescents, indicated that 40.9% used EC once a day and the prevalence of current dual users was 5.2% or 0.17 million [10]. In the United States, the increase in polytobacco use was significant only for those below 25 years old [3]. There is evidence that EC is increasingly becoming a gateway for non-users to become cigarette smokers among adolescents [2]. Among polytobacco users, waterpipe tobacco such as shisha is becoming more prevalent [5, 7–9]. EC and Shisha use are perceived to be trendy, less harmful than cigarette smoking and is socially acceptable among peers [11, 12]. The popularity of EC and other new tobacco products have led to increasing numbers of dual and polytobacco users (more than two tobacco products used concurrently) [13].

A few factors have been linked to higher risks of sole, dual and polytobacco use, notably demographic variables such as gender, age, ethnicity, income and smoking intensity. Worldwide, males are more at risk of smoking behavior compared to females [14–17], even though the gap is smaller in European countries compared to Asia [18]. This phenomenon has been attributed to social stereotypes of smoking as being “unfeminine” [19]. Early adolescents are at less risk of smoking compared to older adolescents [2, 20–22]. Meanwhile, the Malay Bumiputera ethnicity has a higher prevalence of smoking compared to other ethnicities [17, 23].

Past studies indicated that lower socioeconomic status (SES) was an important predictor of tobacco smoking among adolescents [1, 24–26]. Green and colleagues’ [1] research revealed that adolescents from low SES households reported increased initiation and escalation of tobacco use. Smokers from the lower SES group also reported a lower intention to quit [27]. Recent research [28–30] posited that the socialization of adolescents from lower income families of origin, such as the modelling or unhealthy lifestyles and health beliefs, could be transmitted intergenerationally to influence the adolescent’s smoking behavior. On the other hand, the uptake of EC and polytobacco use were highly associated with higher familial and personal income among smokers [22, 31].

The objective of this study was therefore to identify the demographic and socioeconomic characteristics of adolescent smokers and its relationships with adolescent smoking status.

## Methods

### Study design

This is a cross-sectional school-based study, conducted from January to February 2018.

### Participants and sampling

Two levels of sampling were employed: schools were selected using stratified random sampling, whereas the students were purposively selected and invited. A total of six out of 89 secondary schools were selected randomly from all zones in the Federal Territory of Kuala Lumpur, with the ratio of two schools for each of the three zones (Bangsar/Pudu, Sentul and Keramat). Student participants were purposively recruited. A total of 422 students who either smoked conventional cigarettes (CC), electronic cigarettes (EC) and/or shisha for the last 30 days, either self-declared or identified by their discipline teachers to participate in the study. Written consent was obtained from participants, parents and schools.

### Instruments

A stop smoking questionnaire which went through a backward-forward translation from English to Bahasa Malaysia was used for data collection. The questionnaire consisted of demographic characteristics (class, age, year of birth, gender, and race), participants’ daily allowance, monthly household income of parents, measurements related to CC/EC status (Have you ever used a CC/EC, even one or two puffs; age of CC/EC initiation; CC brand and preferable flavor of EC). The smoking status of adolescents was determined based on the question, “Have you ever used: 1. Conventional cigarettes only, 2. Electronic cigarettes only, and 3. A combination of conventional and electronic cigarettes?” The adolescents who answered using only conventional cigarettes were categorized as sole CC users, only electronic cigarettes as sole EC users, and CC and EC use as dual users [32]. The Hooked on Nicotine Checklist (HONC) [33, 34] questionnaire used to determine the onset and strength of tobacco dependence of CC and EC by adding the EC element to the original version. A HONC score of ≥1 indicates a participant is hooked to smoking. The Malay translated HONC reported a Cronbach’s α =0.924.

Exhaled CO level was used to objectively identify the participants’ smoking status. Those with CO level of 4 to 6 ppm were categorized as light smokers and ≥ 7 ppm as regular smokers [2].

### Procedures

Identified participants were screened using exhaled CO to identify their smoking status in addition to self-report for any tobacco use. The participants answered the self-administered questionnaire for approximately 25–30 min. To ensure confidentiality, the participants were informed that there will be no identifiers recorded, only group data will be analyzed and the results will not be disclosed to their parents, teachers or peers. Based on the questionnaire responses, the participants were categorized into sole CC, sole EC, or dual (CC and EC) user categories.

### Statistical analysis

Descriptive and categorical variables were summarized as frequencies and percentages. Chi-square test was used to assess the relationship between sociodemographic, socioeconomic and adolescent smoking status. A multivariate multinomial logistic regression was employed to model the relationship between the predictors and the three smoking statuses (sole CC, sole EC, and dual tobacco users), with dual users as the reference. Household monthly income (with MYR > 4000 as the reference group), HONC (≥1 as reference), CO (≥7 ppm as reference), daily pocket allowance (MYR ≥10 as reference), gender (female as reference), ethnicity (non-Bumiputera as reference), part-time working status (not working part-time as reference), and age (17 years old as reference) were entered as predictors. All analyses were performed using IBM SPSS v.20 (Chicago, IL: SPSS Inc.) software.

## Results

### Demographics, socioeconomic characteristics and smoking status

Of the 422 adolescents (*M* age = 15.58, *SD* = 1.24), 90.3% were males and 69.4% were from the upper form (Form 3 onwards/> 15 years old). The majority were of Malay ethnicity (88.6%) and from the monthly household income group of between MYR1,001–4000 per month (82.2%). More than half of the participants initiated smoking between 13 to 17 years old (59.0%). Most adolescents (89.3%) had a daily pocket allowance of less than MYR10 per day and 12% reported working part-time. More than half reported that they were single users, with a total of 308 (73.0%) participants reporting EC use. The mean CO reading was 2.14 ppm with 78.0% of adolescents scoring more than 0 on the HONC. The response rate of the study was 100% because there were no refusers (Table 1).

**Table 1:** Demographic and socioeconomic characteristics of study participants (*N* = 422)

Characteristic	n (%)
Form	
Form 1	35 (8.4)
Form 2	94 (22.3)
Form 3	90 (21.3)
Form 4	125 (29.6)
Form 5	78 (18.4)
Age (Years), (M = 15.3, SD = 1.24)	
13	34 (8.1)
14	93 (22.0)
15	90 (21.3)
16	123 (29.1)
17	82 (19.4)
Initiation age of smoking (Years)	
< 7	5 (1.2)
7–12	168 (39.8)
13–17	249 (59.0)
Gender	
Male	381 (90.3)
Female	41 (9.7)
Ethnicity	
Bumiputera	331 (78.4)
Non-Bumiputera	91 (21.6)
Daily Pocket Allowance (MYR)	
< 2	21 (5.0)
2- < 5	180 (42.7)
5- < 10	176 (41.7)
≥ 10	45 (10.7)
Household Monthly Family Income (MYR)	
≤ 1000	27 (6.4)
1001–4000	347 (82.2)
≥ RM 4000	48 (11.4)
Working After School (Student)	
Yes	51 (12.1)
No	371 (87.9)
Smoking Status	
Sole CC	114 (27.0)
Sole EC	178 (42.2)
Dual user (CC and EC)	130 (30.8)
HONC (M = 2.65, SD = 2.307)	
Not Hooked (< 1)	90 (21.3)
Hooked (≥1)	332 (78.7)
CO ppm (M = 2.1, SD = 2.682)	
Non-Smoker (1–3)	337 (79.9)
Light Smoker (4–6)	60 (14.2)
Regular Smoker (≥7)	29 (6.9)

*CO* carbon monoxide, *ppm* parts per million, *SD* standard deviation, *HONC* Hooked on Nicotine Checklist

### Characteristics associated with smoking status

Table 2 shows that smoking among adolescents was significantly associated with the upper form (69.4%, *p* < 0.001), age 15 years and above (69.8%, *p* < 0.001), Bumiputera ethnicity (78.2%, *p* < 0.001) and males (90.4%, *p* < 0.001). Smoking status was significantly associated with adolescents with monthly household income of less than RM4,000 (88.6%, *p* = 0.03) and part-time workers (12.1%, *p* = 0.006) but was not significantly related to daily pocket allowance. Household income was also statistically related to smoking initiation age (*p* = 0.033). Adolescents with HONC reading of ≥1 (78.5%, *p* < 0.001) were also related with smoking status even though their CO level < 4 ppm (79.9 *p* < 0.001).

**Table 2:** The association between demographic and socioeconomic characteristics with smoking status (*N* = 422)

Characteristic	Sole CC (*n* = 114)	Sole EC (*n* = 178)	Dual Users (CC and EC) (*n* = 130)	Chi-Square *P*-value
Form				< 0. 001
Form 1	6	20	9	
Form 2	34	36	24	
Form 3	36	16	38	
Form 4	31	61	33	
Form 5	7	45	26	
Age				< 0. 001
13	6	19	9	
14	34	35	24	
15	36	18	36	
16	31	58	34	
17	7	48	27	
Gender				< 0. 001
Male	111	164	106	
Female	3	14	24	
Ethnicity				< 0. 001
Bumiputra	106	115	110	
Non-bumiputra	8	63	20	
Daily Pocket Allowance (MYR)				0.305
< 2	10	6	2	
2- < 5	52	78	38	
5- < 10	42	74	42	
≥10	10	20	8	
Household Monthly Family Income (MYR)				0.005
1000 and below	15	5	7	
1001-RM 4000	91	148	108	
4000 and above	8	25	15	
Part-time Worker				0.003
Yes	22	11	18	
No	92	167	112	
HONC				< 0. 001
Not Hooked (< 1)	10	67	13	
Hooked (≥1)	104	111	117	
CO ppm				< 0. 001
Non-Smoker (1–3)	75	164	98	
Light Smoker (4–6)	28	11	21	
Regular Smoker (≥7)	11	3	11	

*CO* carbon monoxide, *ppm* parts per million, *SD* standard deviation, *HONC* Hooked on Nicotine Checklist

However, results showed that adolescent smoking status was not significantly associated with daily pocket allowance and HONC level.

### Risk factors for sole CC and EC smoking

Results from the multivariate multinomial logistic regression indicated that higher risks for being sole CC users were being male (OR 9.887, 95% CI 2.737, 35.721), aged 15 years old (OR 3.844, 95% CI 1.349, 10.949) and aged 16 years old (OR 3.385, 95% CI 1.199, 9.551). Meanwhile, higher risks for being sole EC users were being male (OR 2.220, 95% CI 1.005, 4.900), not being hooked on smoking (OR 3.951, 95% CI 1.862, 8.387) and non-smoker status based on CO reading of 1–3 ppm (OR 5.777, 95% CI 1.407, 23.716). Meanwhile, Bumiputeras had a lower risk of sole EC smoking (OR 0.467, 95% CI 0.219, 0.994) (Table 3).

**Table 3:** Modelling the adjusted odds between CC or EC versus Dual User using multivariate multinomial logistic regression (*N* = 422)

Smoking Status	Sole CC	Sole EC
	OR	OR
	(95% CI)	(CI 95%)
Household Monthly Income (MYR)		
1000 and below	5.154 (0.407–65.216)	0.464 (0.025–8.598)
1001–4000	2.034 (0.297–13.933)	1.840 (0.409–8.272)
4000 and above^a^	1.000	1.000
HONC		
Not Hooked (< 1)	1.252 (0.474–3.303)	3.951 (1.862–8.387) ***
Hooked (≥1) ^a^	1.000	1.000
CO (ppm)		
Non-Smoker (1–3)	0.689 (0.254–1.872)	5.777 (1.407–23.716)
Light Smoker (4–6)	1.308 (0.420–4.074)	1.968 (0.399–9.704)
Regular Smoker (≥7) ^a^	1.000	1.000
Daily Pocket Allowance (MYR)		
< 2	0.465 (0.030–7.140)	2.930 (0.140–61.196)
2- < 5	0.645 (0.101–4.099)	0.910 (0.196–4.231)
5- < 10	0.423 (0.068–2.629)	0.955 (0.215–4.232)
≥10^a^	1.000	1.000
Gender		
Male	9.887 (2.737–35.721) ***	2.220 (1.005–4.900) **
Female^a^	1.000	1.000
Ethnicity		
Bumiputera	1.542 (0.554–4.293)	0.467 (0.219–0.994) **
Non-Bumiputera^a^	1.000	1.000
Part-time Worker		
Yes	1.561 (0.723–3.374)	0.428 (0.179–1.021)
No	1.000	1.000
Age		
13	3.049 (0.848–10.968)	1.107 (0–0)
14	3.56 (3.56–3.56)	7.368 (0–0)
15	3.844 (1.349–10.949) **	0.384 (0.159–0.930)
16	3.385 (1.199–9.551)**	1.542 (0.721–3.299)
17	1.000	1.000

Reference group: Dual user which includes CC and EC

*** *p* < 0.001. ** *p* < 0.01

^a^ Reference Group

## Discussion

This study revealed that Malaysian adolescents in Kuala Lumpur who were male and aged 15 and 16 years were at increased risks of sole CC smoking. Meanwhile, males, those who are not hooked on smoking and with a non-smoker CO reading were at increased risks of sole EC smoking. Finally, Bumiputeras were at less risk of EC smoking.

The findings showed being male increased the risk of smoking CC by nearly 10 folds while the risk of smoking EC was more than 2-fold compared to females. This is in line with previous studies from Malaysia [14, 17] and abroad [16], which found a higher prevalence of smoking in males compared to females. Asian males are also at a significantly greater risk of smoking than females, who were between 7 and 15 times less likely than males to report any smoking behavior [18]. This may be explained by the prevalent culture which is more accepting of male smokers compared female smokers [19]. However, the risk of smoking EC is lower compared to the risk of smoking CC for males. This is perhaps the EC smoking trend is less stigmatized compared to smoking CC among females. In addition, smoking EC is perceived as a trendy behavior and is more acceptable among adolescents [6]. EC offers a variety of flavours (vanilla, chocolate) which could attract more females and adolescents to try and become hooked.

The risk of 15- and 16-year-old adolescents reporting being sole CC users was higher compared to 17-year-olds, which was not consistent with findings from previous studies [14, 15, 21], where older adolescents were at a higher risk of smoking compared to those who were younger. This might be related to the Sijil Pelajaran Malaysia public examination (equivalent to O-Levels), which may motivate 17-year-olds to be more concerned about their studies. In addition, these older adolescents may have a higher awareness of the health consequences of smoking as they approach their examination.

An interesting finding is that Bumiputera adolescents are at a lower risk of smoking EC. This might be related to their lower SES which did not allow them to buy and maintain EC use. In this study a majority of respondents belonged to the family income of <MYR4000 and EC is related to usage among those with higher SES [22]. This is also in line with previous Malaysian studies which indicated CC smoking is preferred among Bumiputeras based on its price and accessibility [17, 23]. In comparison, Chinese adolescents reported being discouraged from smoking CC and perceived smoking CC as an unhealthy behavior due to receiving similar health-related messages from their elders [24], therefore may choose EC as their smoking preference.

The findings showed that adolescent smoking status was not predicted by household income, working part-time and daily allowance. This is inconsistent with past studies where socioeconomic status and personal income were significant predictors of smoking risk [27–31]. This may be due to the low rate of adolescents who report working part-time (12%), as they may be reluctant to report having a job, as working is prohibited by the law for those under 18 years in Malaysia. In addition, SES may be a weaker predictor of smoking status compared to gender and age. This is because even though Malaysia is one of the countries that implement cigarette taxes, the price of cigarettes in Malaysia is relatively lower [15] due to the the rampant sale of illicit cigarettes [10]. Therefore, the affordability of cigarettes may only minimally affect smoking behavior among adolescents from the lower SES group.

EC appears to be a popular smoking preference among adolescents, with 73% of them reporting EC use in this study. The regulation of EC varies across countries. Presently, in Malaysia, ECs are not banned but are regulated with only nicotine-free e-liquids permitted to be sold by vendors [6]. However, the products are widely available and accessible to youth [17]. There is no barrier to purchasing EC and they are openly available in online stores, roadside stalls/night markets at a low price [10]. This situation contradicts the WHO Framework Convention on Tobacco Control (FCTC), of which Malaysia is a member since September 2005 [17]. There is also the perception that smoking CC poses higher health risks compared to smoking EC. However, extant literature revealed that smoking EC exposed smokers to residual risks due to a lack of control in quality and standards [35]. Therefore, health promotion programs need to address the gap of knowledge in public regarding the relative risks of smoking CC and EC.

Interestingly, the finding reveals that adolescents were also more likely to report using tobacco products even though they were categorized as non-smokers based on their CO level of < 4 ppm. This could be due to the failure to capture smoking status through exhaled CO in spite of self-reported smoking status by the adolescents. Nevertheless, the validity of self-reported smoking has been indicated in a Jordan study, where adolescents tended to be truthful when confidentiality is assured [36]. In addition, adolescents who were very light smokers usually smoke fewer than 6 cigarettes per day [37] and tended to buy single loose cigarettes [38]. The light smokers normally have a lower reading of exhaled carbon monoxide. Furthermore, they may have smoked the last cigarette more than 8 h prior to the breath test at school, hence CO level may have returned to normal. Since 49% of adolescents were sole EC users, this also contributes to the low expired CO concentration observed [39].

### Limitations and strengths

To our best knowledge, this the first study in this region to examine the relationship between demographics, socioeconomic status and adolescent smoking status within multiple groups of tobacco types such as sole EC, sole CC, dual users. However, it has a few limitations. This study was conducted in the Federal Territory of Kuala Lumpur and therefore may not be generalizable to other states in Malaysia. The authors were not able to validate EC use due to financial constraints and therefore depended on the self-report of the participants, unlike CC use which was validated using CO reading. This may lead to inconsistency in the smoking status.

## Conclusions

Adolescents’ smoking status was found to be associated with age, gender, ethnicity, exhaled CO level and being hooked to smoking. These findings are important as they single out those who are most at risk of smoking, and this information provides an indication for targeting specific demographic groups in smoking cessation programs for adolescents.

## Abbreviations

B40: Bottom 40%
CC: Conventional cigarette
CO: Carbon monoxide
DU: Dual users
EC: Electronic cigarette; Malaysia
HONC: Hooked-on Nicotine Checklist
M40: Middle 40%
MMLR: Multivariate multinomial logistic regression
PPM: Parts Per Million
SD: Standard Deviation
SES: Socioeconomic Status
SPSS: Statistical Package for Social Sciences
T20: Top 20%
TECMA: Tobacco & E-Cigarette Survey among Malaysian Adolescents
WHO FCTC: WHO Framework Convention on Tobacco Control
